# Supplementary material for: WordCommentsAnalyzer: A windows software tool for qualitative research
Source: F1000Res. 2018 Sep 4;7:536. Originally published 2018 May 3. [Version 2] doi: 10.12688/f1000research.14819.2 (PMC6137411; doi:10.12688/f1000research.14819.2)
Supplement: Supplementary file 3 [file f1000research-7-17614-s0002.tgz › b2a79496-4ada-47c4-9682-a99ce867836d.zip!/Eight Iranian Health Journals/Yafteh _18.docx]

© Yafteh  . This work is licensed under CC BY 4.0 (https://creativecommons.org/licenses/by/4.0/).

Volume & Issue: دوره 15 - شماره 5

Title: توزیع پلی‌مورفیسم‌های ApoE در جمعیت ‌لر

http://yafte.lums.ac.ir/article-1-1488-fa.html

مقدمه: آپولیپوپروتئینE (ApoE) در استعداد ابتلا به انواع مختلف بیماری‌ها مشارکت دارد. هدف این مطالعه آنالیز ژنوتیپی ApoE در قوم لر برای اولین بار بود. مواد و روش‌ها: ‌در این مطالعه 100 فرد سالم غیرخویشاوند لر از نظر پلی‌مورفیسم‌های ApoE به روش واکنش زنجیره‌ای پلیمراز بر اساس پلی‌مورفیسم طول قطعه محدود شونده (PCR-RFLP) تایپ گردیدند. در نهایت، فراوانی ژنوتیپی و آللی پلی‌مورفیسم‌های ApoE در جمعیت لر با جمعیت ایرانی مقایسه شد. یافته‌ها: شش ژنوتیپ ApoE و تمام پلی‌مورفیسم‌های ApoE در جمعیت لر مشاهده گردیدند. ژنوتیپ با فراوانی 48٪ شایع‌ترین ژنوتیپ در جمعیت لر بود. شایع‌ترین آلل در جمعیت لر نیز آلل بود. بحث و نتیجه‌گیری: نتایج نشان می دهد که فراوانی پلی‌مورفیسم‌های ApoE در جمعیت لر دارای ویژگی‌های کلی گزارش شده در جمعیت‌ ایرانی می‌باشد، ولی با کاهش یا افزایش برخی فراوانی‌ها، هنوز هم منحصر به فرد است.

Title: عوامل پیشگویی کننده الگوهای ارتباطی مادر و کودک در مراجعین به بیمارستان شهید مدنی شهر خرم آباد در سال 1391

http://yafte.lums.ac.ir/article-1-1489-fa.html

مقدمه: کیفیت ارتباط مادر وکودک نقش بسزایی درشکل گیری شخصیت، عملکرد اجتماعی و بهداشت روان کودک درآینده دارد. این پژوهش با هدف بررسی الگوهای ارتباطی مادر و کودک و عوامل پیشگویی کننده آن طراحی شده است. مواد و روش‌ها: در این مطالعه مقطعی 110 مادر مراجعه کننده به بیمارستان کودکان شهر خرم آباد به روش نمونه گیری متوالی وارد مطالعه شدند. ابراز گردآوری اطلاعات پرسشنامه ارزیابی رابطه والد و کودک (MCRE) بود. ابتدا نقطه نظرات مادران را در 4 حیطه پذیرش فرزند، بیش حمایتگری، سهل‌گیری افراطی و طرد فرزند مورد بررسی قرار گرفت. سپس با توجه به نقطه برش هرحیطه، نوع الگوی ارتباطی تعیین شد. از آزمون دقیق فیشر و تحلیل رگرسیون خطی جهت تجزیه و تحلیل اطلاعات استفاده شد. بحث و نتیجه‌گیری: بالا بردن سطح آگاهی مادران از طریق برگزاری کارگاه‌های آموزشی می‌تواند مهارت پرورش صحیح فرزند را افزایش داده و منجر به پرورش مهارت‌های اجتماعی و کاهش مشکلات رفتاری کودکان در آینده شود. یافته‌ها: میانگین سنی مادران 64/6± 6/28 سال بود. الگوی ارتباطی4/66% مادران بیش حمایتگری و 1/19% آنها سهل انگاری افراطی بود. 8/12% از الگوی پذیرش فرزند و 8/1% ازالگوی ارتباطی طرد فرزند پیروی می‌کردند. بین متغیرهای سن، میزان تحصیلات، وضعیت تاهل و نوع بارداری و همچنین محل سکونت ماردران با نوع الگوی ارتباطی آنها ارتباط آماری معنادار دیده شد (05/0< P). در تحلیل رگرسیون خطی مهمترین فاکتور پیشگویی کننده نوع الگوی ارتباطی، تک والدی بودن و بارداری برنامه ریزی نشده بود.

Title: بررسی شیوع بیماری مادرزادی قلبی وانواع آن در نوزادان متولد شده در شهر خرم آباد

http://yafte.lums.ac.ir/article-1-1490-fa.html

مقدمه: بیماری های مادرزادی قلب شایع ترین ناهنجاری مادرزادی در نوزادان است. هدف ازاین مطالعه بررسی شیوع بیماری مادرزادی قلبی وانواع آن در نوزادان متولد شده در شهر خرم آباد بود. بحث و نتیجه‌گیری: در این مطالعه نقص دیواره بین بطنی و نقص دیواره بین دهلیزی شایع ترین ناهنجاری های مادرزادی قلب بودند و فراوانی جنسیت نوزاد پسر تقریباً دو برابر نوزاد دختر بود. یافته‌ها: از 43195 نوزاد متولد شده 177 مورد بیماری مادررزادی قلبی داشتند و میزان شیوع بیماری های مادرزادی قلبی در هر 1000 تولد زنده در این مطالعه 2/4 بود. در این مطالعه نقص دیواره بین بطنی 44 درصد و نقص دیواره بین دهلیزی 21 درصد شایع ترین ناهنجاری های مادرزادی قلب بودند. 8/63 درصد از نوزادان پسر (005/0P=)، 4/80 درصد از نوزادان ترم (001/0< P)، 5/93 درصد از نوزادان بدون سیانوز، نسبت فامیلی والدین در 28 درصد، سابقه بیماری های مادرزادی قلب در والدین در 3/10 درصد، سابقه بیماری دیابت در3/9 درصد مادران و 6/5 درصد از نوزادان آنومالی سایر اعضا از جمله شکاف کام و لب داشتند. مواد و روش‌ها: مطالعه حاضر از نوع توصیفی- مقطعی و روش نمونه گیری به صورت سر شماری بود. تمام نوزادان متولد شده در شهر خرم آباد از اول اردیبهشت سال1385 تا پایان فروردین 1390 که در اکو کاردیوگرافی آنها بیماری های مادرزادی قلب تشخیص داده شد مورد مطالعه قرار گرفتند. متغیرهای مورد مطالعه شامل جنس، نارسی نوزاد، سیانوز، آنومالی سایر اعضاء، سن بالای 40 سال مادر، نسبت فامیلی پدر و مادر، مصرف دارو در دوران بارداری، سابقه بیماری در مادر و نوع ناهنجاری قلبی بود. اطلاعات مربوط به بیماران از طریق پرسشنامه جمع آوری گردید. داده ها با استفاده از نرم افزار SPSS مورد تجزیه و تحلیل آماری قرار گرفتند.

Title: شیوع کودک آزاری در دانش آموزان مقطع راهنمایی خرم آباد در سال 1390

http://yafte.lums.ac.ir/article-1-1491-fa.html

مقدمه: کودک آزاری یک مشکل جهانی است و به اشکال مختلف رخ می‌دهد که عمیقاً ریشه در فرهنگ، عملکرد اقتصادی و اجتماعی دارد. کودک آزاری رفتاری است که باعث آزار جسمی، ذهنی، عاطفی یا جنسی کودک می‌شود که در اثر این اعمال، سلامتی و آسایش و تحصیل کودک آسیب می‌بیند. با توجه به اهمیت موضوع، این بررسی جهت تعیین میزان شیوع کودک‌آزاری در کودکان مدارس راهنمایی خرم آباد در سال 1390 طراحی و اجرا گردید. بحث و نتیجه‌گیری: نتایج نشان داد که سوء رفتار با کودکان در خانواده‌ها شایع است. لذا ضرورت پایش کودکان، تدوین قوانین حمایت از کودکان و طرح و اجرای برنامه های آموزشی پیشگیری می‌تواند در کاهش کودک آزاری مفید باشد. یافته‌ها: نشان داد که میانگین سنی نمونه‌ها 04/1±36/13 بود. 4/5٪ نمونه‌ها به صورت همیشه تحت آزار جسمی و بیشترین آزار جسمانی سیلی خوردن بود. 3/7، تحت آزار عاطفی به طور همیشگی بودند و 5/5٪ بی توجهی را به صورت همیشگی تجربه کرده بودند. آزمون آمار ارتباط معناداری بین آزارها با تحصیلات، شغل، اعتیاد و طلاق والدین نشان داد. مواد و روش‌ها: مطالعه فوق توصیفی مقطعی که 907 دانش آموز مقطع راهنمایی به روش تصادفی انتخاب شدند. ابزار گردآوری داده‌ها پرسشنامه ای چند قسمتی شامل اطلاعات فردی کودک و والدین، پرسشنامه آزار جسمی عاطفی و بی توجهی بود. روایی و پایایی ابزار با روایی محتوا و آزمون مجدد کسب شد اطلاعات با استفاده از نرم افزار spss 19 تجزیه تحلیل شدند.

Title: طراحی و ساخت سازه‌ ژنی نوترکیب بیان کننده ژن حفاظت کننده سلولی

http://yafte.lums.ac.ir/article-1-1492-fa.html

بحث و نتیجه‌گیری: ساخت سازه نوترکیبMT1-pccDNA3.1 و ورود موفق آن به درون سلول‌های بنیادی مزانشیمی می‌تواند به عنوان یکی از راهبُردهای محافظت سلول‌های پیوندی از مرگ سلولی پیشنهاد گردد و ممکن است بتواند راه کاری جدید در راستای ارتقاء کارآمدی سلول درمانی بعد از پیوند را فراهم آورد. مواد و روش‌ها: سلول‌های بنیادی مزانشیمی از مغز استخوان انسان جداسازی و توسط روش فلوسایتومتری تأیید شدند. توالی کامل cDNA ژن MT1 جداسازی شد و برای وارد شدن در وکتور پلاسمیدی pcDNA 3.1 مورد استفاده قرار گرفت. سازه ژنی نوترکیب ایجاد شده، به روش لیپوفکشن وارد MSCs گردید. بیان MT1 توسط RT-PCR و لکه‌گذاری وسترن پایش شد. یافته‌ها: ژن MT1 انسانی نوترکیب با موفقیت در وکتور pcDNA کلون گردید و درست قرار گرفتن ژن در قالب صحیح درون وکتور به وسیله تعیین توالی DNA تأیید شد. افزایش بیان MT1 در MSCs به وسیله روش های RT-PCR و لکه‌گذاری وسترن مورد تأیید قرار گرفت. این نتایج نشان دهنده بیان موقت MT1 در MSCs بود. مقدمه: دست‌ورزی ژنتیکی یک راهبرد مؤثر برای حفاظت سلول‌ها در برابر آسیب‌های محیطی و بالا بردن توانمندی آنان در استفاده‌های درمانی است. به منظور جلوگیری از عوارض ناخواسته‌ای همچون ایجاد بدخیمی‌ها، دست‌کاری ژنتیکی و افزایش بیان ژن‌های حاصل از آن می‌بایست به صورت موقت باشد. هدف از انجام این پژوهش، کلونینگ و افزایش بیان ژن محافظ سلولی "متالوتیونین یک" (MT1) انسانی به صورت موقت در سلول‌های بنیادی مزانشیمی (MSCs) با استفاده از سیستم بیانی پلاسمیدی و استفاده از وکتور pcDNA 3.1 بود. این افزایش بیان به منظور افزایش مقاومت سلول‌های مزانشیمی نسبت به آسیب های محیطی و ... است.

Title: بررسی فراوانی نتایج مثبت تست تحریک صوتی در ارزیابی سلامت جنین در زنان باردار با NST غیر واکنشی

http://yafte.lums.ac.ir/article-1-1493-fa.html

مقدمه: آزمون غیراسترسی(NST) یکی از رایج ترین ابزارهای ارزیابی سلامت جنین می باشد که نتایج آن به صورت واکنشی و غیرواکنشی تعبیر می شود. از محدودیت های این آزمون بالا بودن موارد غیرواکنشی کاذب می باشد. مطالعات مختلف همراهی تحریک صوتی ارتعاشی با NSTرا جهت کاهش موارد غیرواکنشی و مدت آزمون پیشنهاد نموده اند. بحث و نتیجه‌گیری: با استفاده از تحریک صوتی ارتعاشی در ارزیابی سلامت جنین می توان با صرفه جویی در وقت، هزینه و پرسنل، سلامتی جنین را زودتر و بهتر تشخیص داد. یافته‌ها: در این پژوهش 5/85% موارد NST غیرواکنشی (47 مورد) با استفاده از تحریک صوتی ارتعاشی به NST واکنشی تغییر یافت. مواد و روش‌ها: این پژوهش از نوع بررسی تست ها به صورت یک گروهی و به روش نمونه گیری تصادفی ساده بود. تعداد نمونه ها 55 خانم باردار با NST و ابزار گردآوری داده ها شامل پرسشنامه و برگ ثبت اطلاعات، دستگاه سونوگرافی و مانیتورینگ جنین، کاغذ الگوهای ضربان قلب، مسواک الکتریکی و ساعت بود. تحریک صوتی ارتعاشی با مسواک الکتریکی به مدت سه ثانیه بالای سرجنین از روی شکم مادر انجام و NST ده دقیقه دیگر ادامه می یافت و سپس نتایج آن ثبت می شد. داده‌های بدست آمده توسط نرم افزار SPSS 19 جمع آوری و با استفاده از آمار توصیفی مورد تجزیه و تحلیل قرار گرفت.

Title: اثر بازی های بومی محلی و بازی های معمول بر رشد مهارت های دستکاری پسران کم توان ذهنی آموزش پذیر

http://yafte.lums.ac.ir/article-1-1494-fa.html

مقدمه: مهارت های دستکاری جزو یکی از زیر شاخه های مهارت های بنیادی است که در بسیاری از اعمال روزمره و فعالیت های ورزشی مورد استفاده قرار می گیرند. هدف این مطالعه مقایسه اثر بازی های بومی محلی با بازی های معمول بر رشد مهارت های دستکاری پسران کم توان ذهنی آموزش پذیر 8 تا 10 ساله شهر گرگان بود. بحث و نتیجه‌گیری: می توان گفت، بازی های بومی محلی منتخب می توانند برنامه مناسب برای رشد مهارت های دستکاری کودکان باشند. یافته‌ها: دو گروه به شکل معنی داری در مهارت های دستکاری پیشرفت داشتند اما پیشرفت گروه بازی های بومی محلی به شکل معنی داری بیشتر از گروه بازی های معمول در تمام مهارت های دستکاری اندازه گیری شده (پرتاب کردن، دریافت کردن، ضربه با پا، ضربه به توپ از پهلو، دریبل ثابت و غلتاندن توپ) بود (05/0>P). مواد و روش‌ها: اطلاعات فردی از طریق پرسشنامه محقق ساخته و هوش کودکان با آزمون وکسلر بررسی شد و در مرحله پیش آزمون، مهارت دستکاری افراد از طریق نسخه دوم آزمون مهارت حرکتی درشت اولریخ سنجش شد. سپس افراد به شکل تصادفی در دو گروه؛ بازی های بومی محلی (15 نفر) و بازی های معمولی (15 نفر) توزیع شدند. بعد از 8 هفته تمرین (سه جلسه در هفته و هر جلسه 45 دقیقه) از هر دو گروه پس آزمون مهارت های دستکاری به عمل آمد. تحلیل داده ها با استفاده از آزمون تی مستقل، تی وابسته و تحلیل واریانس با اندازه گیری مکرر در سطح معنی داری (05/0 =α) انجام گرفت.

Title: اثرات برنامه توانبخشی قلب بر ظرفیت ورزشی و عوامل خطرزای کرونری بیماران جراحی شده بای‌پس کرونری 45 تا 65 سال

http://yafte.lums.ac.ir/article-1-1495-fa.html

مقدمه: بیماری های قلبی- عروقی شایعترین علت مرگ و ناتوانی انسان محسوب می شود. یک سوم علت مرگ و میر در ایران، بیماری های کرونری قلب می باشد. از این رو، در بررسی حاضر اثرات برنامه توانبخشی قلبی بر ظرفیت ورزشی و عوامل خطرزای کرونری بیماران جراحی شده بای پس کرونری (CABG) 45 تا 65 سال ارزیابی گردید. بحث و نتیجه‌گیری: برنامه توانبخشی قلب مطالعه حاضر اثر معناداری بر ظرفیت ورزشی و پروفایل برخی عوامل خطرزای کرونری بیماران جراحی شده بای پس کرونری 45 تا 65 سال را نشان داد و می تواند به عنوان یک برنامه مناسب برای این بیماران در نظر گرفته شود. یافته‌ها: تجزیه و تحلیل آماری با آزمون t مستقل نشان داد که در انتهای برنامه تفاوت معناداری بین درصد تغییرات VO2max، BMI، WHR، SBP، DBP، TC، LDL، LHR دو گروه وجود دارد (05/0P<)، اما بین درصد تغییرات FBS، HDL، BFP دو گروه تفاوت معناداری وجود نداشت. مواد و روش‌ها: با انتخاب تصادفی 36 بیمار جراحی شده به روش CABG بیمارستان شهید بهشتی همدان، 18 آزمودنی با میانگین سنی 26/6±57/54 سال و میانگین وزن 83/10±14/72 کیلوگرم برای گروه آزمون( مشارکت در برنامه توانبخشی) و 18 آزمودنی با میانگین سن 75/4±64/57 سال و میانگین وزن 12±5/76 کیلوگرم برای گروه شاهد (عدم شرکت در هرگونه برنامه توانبخشی) بر اساس معیارهای ورود و خروج در بررسی حاضر شرکت کردند. برنامه توانبخشی قلبی یک برنامه 8 هفته‌ای شامل گرم کردن، ‌ورزش های هوازی و سرد کردن بود. متغیرها شامل اندازه های آنتروپومتریک، ‌ظرفیت ورزشی (VO2max) و عوامل خطرزای کرونری از جمله شاخص توده بدنی (BMI)، نسبت دور کمر به دور باسن (WHR)، فشار خون سیستولیک (SBP)، فشار خون دیاستولیک (DBP)، قند خون ناشتا (FBS)، کلسترول تام (TC)، تری گلیسرید (TG)، لیپو پروتئین کم چگال (LDL)، لیپو پروتئین پر چگال (HDL)، نسبت LDL بر HDL (LHR)، درصد چربی بدن (BFP) در اولین و آخرین هفته برنامه در هر دو گروه تعیین شدند.

Title: بررسی کیفیت آب رودخانه خرم‌رود خرم‌آباد با شاخص کیفیت آب (NSFWQI) و پهنه بندی آن با سامانه اطلاعات جغرافیایی (GIS)

http://yafte.lums.ac.ir/article-1-1496-fa.html

مقدمه: رودخانه‌ها جزء کوچکی از آب‌های جاری جهان و به‌عنوان یکی از منابع اساسی تأمین آب برای مصارف گوناگون از جمله کشاورزی، شرب و صنعت مطرح می‌باشند. هدف از این مطالعه بررسی کیفیت آب رودخانه خرم‌رود خرم‌آباد با استفاده از شاخص کیفیت آب (NSFWQI) و سامانه اطلاعات جغرافیایی (GIS) بوده است. مواد و روش‌ها: در این مطالعه مقطعی، پارامترهای کیفی مورد نیاز جهت محاسبه شاخص (NSFWQI) شامل pH، اکسیژن محلول، کل جامدات، اکسیژن مورد نیاز بیوشیمیایی، کدورت، دما، فسفات، نیترات و کلیفرم مدفوعی در شش ایستگاه انتخابی به مدت شش ماه از سال 1391 با استفاده از روش استاندارد مورد اندازه‌گیری قرار گرفت. پهنه‌بندی مسیر رودخانه با استفاده از نرم‌افزار GIS صورت گرفت. یافته‌ها: با توجه به نتایج حاصل از این مطالعه، بالاترین کیفیت آب در ایستگاه شماره یک با میزان عددی شاخص (NSFWQI) معادل 82 (آب‌های با کیفیت خوب) در مردادماه و آبان‌ماه و بدترین کیفیت آب در ایستگاه شماره شش با میزان عددی شاخص (NSFWQI) معادل 42 (آب‌های با کیفیت بد) در شهریورماه و آبان‌ماه گزارش شد. هرچه از ایستگاه اول به سمت ایستگاه‌های آخر حرکت می‌کنیم آلودگی آب بیشتر و از کیفیت آن کاسته می‌شود. بحث و نتیجه‌گیری: بر اساس نتایج بدست آمده، شاخص کیفیت آب (NSFWQI) شاخص خوبی جهت مشخص نمودن اثر منابع آلوده-کننده بر روی آب رودخانه می‌باشد. نتایج نشان داد که بر اساس میانگین شاخص مورد مطالعه کیفیت آب رودخانه در ایستگاه اول خوب، ایستگاه‌های دوم، سوم و چهارم متوسط و در ایستگاه‌های پنجم و ششم بد می‌باشد. نتایج فوق امکان تصمیم‌گیری در خصوص پایش و کنترل منابع آلوده‌کننده آب رودخانه و استفاده مؤثر از آن را جهت مصارف مختلف برای مسئولین ذی‌ربط فراهم می‌آورد.

Title: بررسی نشانه های اضطراب در آزمون نقاشی آدمک کودکان 10 ساله شهر خرم آباد

http://yafte.lums.ac.ir/article-1-1497-fa.html

مقدمه: اختلالات اضطرابی از جمله اختلالات شایع در کودکان می باشند که می توانند برای کودکان بسیاری از اختلالات دیگر از جمله اعتماد به نفس پایین، کناره گیری اجتماعی، مهارتهای اجتماعی ناکافی و مشکلات تحصیلی را نیز به دنبال داشته باشند. لذا با توجه به اهمیت این اختلالات بر آن شدیم تا به تعیین نشانه های اضطراب در آزمون نقاشی آدمک کودکان شهر خرم آباد بپردازیم. بحث و نتیجه‌گیری: با توجه به نتایج پژوهش حاضر و اهمیت تشخیص اختلالات اضطرابی در دوران کودکی و تاثیری که در دورانهای دیگر زندگی بر جای می گذارند ،پیشنهاد می شود که به این نشانه ها در نقاشی کودکان توجه بیشتری شود و با تشخیص به موقع این کودکان از تاثیرات سوء اضطراب در آینده بر روی آنها جلوگیری به عمل آید. یافته‌ها: نشانه های معنا دار(05/0< P)در کودکان گروه آزمون (مضطرب) عبارت بودند از: چشم های کوچک، خطوط ترسیمی بسیار پررنگ، خطوط ترسیمی موازی سایه دار و چهار خانه، خط خطی کردن و پاک کردنهای مکرر، وضع بدنی نا متعادل، استفاده از رنگ سیاه، حالت صورت آدمک مضطرب و نگران، سر کوچک، ساقها طویل، پاها بزرگ، بازوها بصورت نامنعطف و افقی و دستها بصورت بسته. همچنین نشانه های اضطراب درآزمون نقاشی آدمک بر حسب جنسیت نیز معنا دار بود. مواد و روش‌ها: در این مطالعه مقطعی 455 نفر از دانش آموزان پایه چهارم دبستان های شهر خرم‌آباد با استفاده از نمونه گیری خوشه ای انتخاب شدند. سپس با استفاده از مقیاس تجدید نظر شده اضطراب آشکار کودکان مورد ارزیابی قرار گرفتند و در نهایت 102 نفر از آنها در غالب دو گروه آزمون (51 نفر از بین کودکانی که نمره بالا در مقیاس ذکر شده کسب کرده بودند) و گروه کنترل (51 نفر از بین کودکانی که نمره پایین در مقیاس ذکر شده کسب کرده بودند) انتخاب و آزمون نقاشی آدمک در بر روی آنها اجراء شد و در نهایت نشانه‌ها با استفاده از آمار توصیفی (میانگین و انحراف استاندارد) و آزمونهای آمار استنباطی (کای دو و آزمون t )به کمک نرم افزار SPSS بررسی شدند.

Title: فریتین سرم و نسبت آنزیم‌های کبدی و توافق آنها با شدت کبد چرب غیرالکلی

http://yafte.lums.ac.ir/article-1-1498-fa.html

مقدمه: کبد چرب غیر الکلی (NAFLD)شایع‌ترین بیماری کبدی است. در این بیماری به دلیل آسیب و کارکرد غیر طبیعی سلول‌های کبدی افزایش سطوح آنزیم‌های کبدی مشاهده می‌شود. در اغلب موارد افزایش سطح فریتین سرم به عنوان پارامتری از وضعیت کارکردی کبد مشاهده می‌شود. هدف مطالعه حاضر تعیین توافق بین آنزیم‌های کبدی و فریتین با درجه اکوژنیسیته کبدی بر اساس سونوگرافی در بیماران NAFLD است. بحث و نتیجه‌گیری: یافته‌های مطالعه حاضر حاکی از توافق بین شدت بیماری کبد چرب با سطح فریتین سرم و نسبت آنزیم‌های کبدی در بیماران NAFLD اما نه با نسبت فریتین به آنزیم‌های کبدی می باشد. یافته‌ها: میانگین فریتین سرم ng/ml25/108±34/126 بود که تفاوت معنی‌دار با شدت اکوژنیسیته کبدی نشان داد )016/0=P (. ارتباط معنی‌داری میان درجه کبد چرب و نسبت آنزیم‌های ALT/ASTوجود داشت (004/0=P) و میزان توافق (عدد کاپا) 3/68% بود، در حالی ‌که توافق معنی‌دار میان نسبت فریتین به این دو آنزیم با شدت کبد چرب نشان نداد. مواد و روش‌ها: مطالعه مقطعی حاضر بر روی 60 بیمار NAFLD تایید شده با اولتراسونوگرافی در تبریز انجام گردید. از کلیه افراد نمونه خون ناشتا تهیه و در سرم آلانین آمینوترانسفراز، آسپارتات آمینوترانسفراز و فریتین اندازه گیری شد. قد و وزن ارزیابی گردید. نسبت آنزیم-های کبدی محاسبه و به سهک ها تقسیم بندی شد.

Title: ارزیابی تغییرات قلب موش های نر نژاد ویستار در پی تمرینات استقامتی

http://yafte.lums.ac.ir/article-1-1499-fa.html

مقدمه: هدف این مطالعه اندازه گیری دقیق پاسخ های قلب به تمرینات استقامتی با استفاده از دو روش m-mode و توزین در موش‌ها و هم چنین ارزیابی تغییرات ناشی از تمرین در قلب و بطن چپ به نسبت وزن بدن، طول استخوان درشت نی و سطح رویه بدن است. بحث و نتیجه‌گیری: به نظر می رسد، برای ارزیابی سازگاری های قلبی ناشی از فعالیت های استقامتی، روش m-mode قابل اعتماد تر است. به علاوه برای نسبی کردن شاخص های وزنی قلب، استفاده از وزن بدن و سطح رویه بدن که با هزینه انرژی بدن در ارتباطند پیشنهاد می شود. یافته‌ها: در مقایسه با گروه کنترل، قطر دیاستولی بطن چپ در گروه تجربی به طور معنی داری افزایش یافته بود. در بین پارامترهای ترکیبی، فقط میانگین های کوتاه شدن کسری و حجم ضربه ای در بین گروه ها به صورت معنی دار متفاوت بود که با ارزیابی نسبی، تفاوت‌ها محسوس تر شد. مواد و روش‌ها: 20 موش (231-209 گرم) به صورت تصادفی به دو گروه کنترل و تجربی تقسیم و در شرایط استاندارد نگهداری شدند. گروه تجربی یک برنامه تمرینی (14 هفته تمرین استقامتی) را اجرا کرد. ابعاد، وزن قلب و بطن چپ به ترتیب به وسیله اولتراسوند و ترازو اندازه گیری و بر اساس طول درشت نی، سطح رویه بدن و هم چنین وزن بدن به صورت نسبی ارزیابی شد. تفاوت های آماری بین دو گروه به وسیله آزمون t مستقل محاسبه و حداقل سطح معنی داری 05/0 در نظر گرفته شد.
